# Taxonomy and phylogeny reveal new species of *Minuticlypeus* (Pallidoperidiaceae, Xylariales) and *Paravamsapriya* (Vamsapriyaceae, Xylariales) from Yunnan-Guizhou Plateau, China

**DOI:** 10.3897/mycokeys.137.195655

**Published:** 2026-07-22

**Authors:** Zu-Quan Yao, Li-Li Liu, Wen-Hao Li, Qing Yang, Nalin N. Wijayawardene, Al-Bandari Fahad Al-Arjani, Abdallah M. Elgorban, Faten Zubair Filimban, Kamran Habib, Qi-Rui Li

**Affiliations:** 1 State Key Laboratory of Discovery and Utilization of Functional Components in Traditional Chinese Medicine & Guizhou Key Laboratory of Microbio and Infectious Disease Prevention & Control, Guizhou Medical University, Guian New District, Guiyang, Guizhou 550004, China High-Value Food from Mushrooms and Bioactive Plants in the Green Economy Value Chain Research Group, The Institute of Biotechnology and Genetic Engineering, Chulalongkorn University Bangkok Thailand; 2 Guizhou International Science & Technology Cooperation Base for Druggability Research of Natural Medicines (The Key Laboratory of Optimal Utilization of Natural Medicine Resources), School of Pharmaceutical Sciences, Guizhou Medical University, Guiyang, Guizhou 561113, China Center for Yunnan Plateau Biological Resources, Protection and Utilization & Yunnan International Joint Laboratory of Fungal Sustainable Utilization in South and Southeast Asia, College of Biology and Food Engineering, Qujing Normal University Qujing China; 3 Department of Entomology and Plant Pathology, Faculty of Agriculture, Chiang Mai University, Chiang Mai 50200, Thailand Botany and Microbiology Department, College of Science, King Saud University Riyadh Saudi Arabia; 4 Engineering Research Center of Health Medicine Biotechnology of Institution of Higher Education of Guizhou Province, School of Biology and Engineering (School of Health Medicine and Modern Industry), Guizhou Medical University, Guian New District, Guiyang, Guizhou 561113, China Center of Excellence in Biotechnology Research (CEBR), DSR, King Saud University Riyadh Saudi Arabia; 5 Center for Yunnan Plateau Biological Resources, Protection and Utilization & Yunnan International Joint Laboratory of Fungal Sustainable Utilization in South and Southeast Asia, College of Biology and Food Engineering, Qujing Normal University, Qujing, China Division of Plant Sciences, Department of Biological Sciences, Faculty of Sciences, King Abdulaziz University Jeddah Saudi Arabia; 6 High-Value Food from Mushrooms and Bioactive Plants in the Green Economy Value Chain Research Group, The Institute of Biotechnology and Genetic Engineering, Chulalongkorn University, Bangkok, Thailand State Key Laboratory of Discovery and Utilization of Functional Components in Traditional Chinese Medicine & Guizhou Key Laboratory of Microbio and Infectious Disease Prevention & Control, Guizhou Medical University Guiyang China; 7 Botany and Microbiology Department, College of Science, King Saud University, Riyadh 11451, Saudi Arabia Guizhou International Science & Technology Cooperation Base for Druggability Research of Natural Medicines (The Key Laboratory of Optimal Utilization of Natural Medicine Resources), School of Pharmaceutical Sciences, Guizhou Medical University Guiyang China; 8 Center of Excellence in Biotechnology Research (CEBR), DSR, King Saud University, Riyadh, Saudi Arabia Engineering Research Center of Health Medicine Biotechnology of Institution of Higher Education of Guizhou Province, School of Biology and Engineering (School of Health Medicine and Modern Industry), Guizhou Medical University Guiyang China; 9 Division of Plant Sciences, Department of Biological Sciences, Faculty of Sciences, King Abdulaziz University, Jeddah, Saudi Arabia Department of Entomology and Plant Pathology, Faculty of Agriculture, Chiang Mai University Chiang Mai Thailand

**Keywords:** Bambusicolous fungi, fungal systematics, karst ecosystem, xylariaceous fungi

## Abstract

Surveys of Xylariales fungi conducted in southwestern China resulted in the collection of several specimens from Yunnan and Guizhou Provinces. Morphological examination, together with multi-locus phylogenetic analyses based on combined ITS, LSU, *rpb2*, and *tub2* sequence data, revealed that these specimens represent two previously undescribed species within *Minuticlypeus* and *Paravamsapriya*. These are introduced here as *Minuticlypeus zunyiensis***sp. nov**. and *Paravamsapriya yunnanensis***sp. nov**. Detailed morphological descriptions, illustrations, and phylogenetic trees clearly delineate these taxa, highlighting their distinct affiliations with existing species.

## Introduction

Xylariales is the second largest order within Xylariomycetidae, comprising 165 genera distributed across 22 families, along with an additional 57 genera placed without any familial placements (Hyde et al. 2024). Members of the order are ecologically important and occur ubiquitously on decaying wood, fallen branches, and trunks. As major saprotrophs, they play a crucial role in the decomposition of lignocellulosic substrates and nutrient cycling in terrestrial ecosystems (Becker and Stadler 2021; Franco et al. 2022). Many species occur as endophytes in healthy plant tissues, where they contribute to plant-microbe interactions, enhance host fitness, and may provide protection against pathogens (Helaly et al. 2018; Franco et al. 2022). Xylariales are also recognized as prolific producers of diverse secondary metabolites with ecological functions in competition, defense, and symbiosis, as well as significant potential for pharmaceutical, agricultural, and biotechnological applications (Helaly et al. 2018; Becker and Stadler 2021). Despite their abundance, the taxonomy of Xylariales remains challenging due to limited molecular data, uncertain teleomorph and anamorph connections, and the polyphyletic nature of taxa with poorly developed stromata, making species delimitation difficult (Samarakoon et al. 2022; Liu et al. 2025). Despite these taxonomic and morphological challenges in accurately identifying species within this group, interest in Xylariales has increased in recent years, largely due to their ecological significance and species diversity (Li et al. 2024; Habib et al. 2025; Liu et al. 2025).

The genus *Minuticlypeus* (Pallidoperidiaceae) was established by Sugita et al. (2024), with *M. discosporus* as the type species. The genus is known only from its sexual morph, characterized by immersed perithecia beneath a small clypeus, surrounded by pseudostromatic tissue; solitary, subglobose ascomata with periphysate conical ostiolar necks; unbranched paraphyses; 8-spored asci with an amyloid, inverted hat-shaped apical apparatus; and unicellular, ellipsoid to oblong brown ascospores, flattened fusiform in side view, enclosed in a mucilaginous sheath with a germ slit extending the full length. The genus is represented by five species found exclusively on bamboo, four of which are reported from Southwestern China (Li et al. 2024; Habib et al. 2025). The genus *Paravamsapriya* (Vamsapriyaceae) was introduced by Samarakoon et al. (2022), with *P. ostiolata* as the type species found on dead bamboo branches. The genus is known only from its sexual morph, characterized by immersed, black ascomata with a yellowish-brown clypeus-like margin; centric, periphysate ostioles; a multi-layered peridium with a thick-walled outer layer and hyaline inner layer; long, septate paraphyses; 8-spored, unitunicate, cylindrical, short-pedicellate asci with an apical ring J−; and hyaline, ellipsoidal to fusiform, aseptate ascospores lacking germ slits. The genus is represented by two species, *P. ostiolata* reported from Thailand (Samarakoon et al. 2022) and *P. nypae* from China (Zhang et al. 2024). *Paravamsapriya* is morphologically similar to *Vamsapriya*, but can be distinguished by the presence of a yellow-black margin around the host surface around the ostioles, an easily detachable peridium, and aseptate ascospores (Samarakoon et al. 2022).

During field surveys of xylariaceous fungi, we collected specimens of *Minuticlypeus* and *Paravamsapriya* from the Yunnan-Guizhou Plateau, China. Morphological examination and multi-locus phylogenetic analyses using ITS, LSU, *rpb2*, and *tub2* sequences confirmed that these specimens represent previously undescribed species, distinct from all known members of their respective genera. This study highlights the richness and largely unexplored diversity of xylariaceous fungi in southwestern China. Continued surveys and integrative studies are essential to document and understand their ecological and taxonomic roles.

## Materials and methods

### Sample collection

Samples were collected during field surveys conducted in August and September 2025 in Kuankuoshui National Nature Reserve, Guizhou Province, and the Xishuangbanna National Nature Reserve, Yunnan Province, China. Both reserves are characterized by warm, humid climatic conditions and well-preserved natural forest ecosystems that support high levels of biodiversity. Kuankuoshui experiences a humid subtropical monsoon climate with a mean annual temperature of approximately 13–20 °C, annual precipitation of 1,100–1,500 mm, and relative humidity generally exceeding 80%. The vegetation is dominated by mixed forest and bamboo forest (Zhou et al. 2024). Xishuangbanna has a tropical monsoon climate with mean annual temperatures ranging from 18–22 °C, annual precipitation of 1,200–2,000 mm, and high year-round humidity. The reserve contains extensive natural tropical seasonal rainforests, tropical monsoon forests, and montane evergreen broad-leaved forests (Zhu 2006). Detailed habitat information, including latitude, longitude, and altitude, was recorded during collection. Samples were transported to the laboratory in sealed bags, air-dried under ambient conditions for subsequent morphological examination and isolation of strains. All examined specimens were deposited in the Herbarium of Guizhou Medical University (**GMB**) and the Herbarium of Cryptogams, Kunming Institute of Botany, Chinese Academy of Sciences (**KUN-HKAS**).

### Morphological characterization and isolation

Macroscopic features (e.g., ostioles, clypeus) were examined using an Olympus SZ61 stereomicroscope. Microscopic characteristics (e.g., ascomata, peridium, paraphyses, asci, ascospores) were observed with a Nikon Ni compound microscope. Photographs were taken using a Canon 700D digital camera attached to both microscopes. The amyloid reaction of the apical apparatus was tested using Melzer’s reagent. Measurements of asci and ascospores were made using Tarosoft Image Framework (v.0.9.0.7). Images were processed with Adobe Photoshop CS6. Pure cultures were attempted via single-ascospore isolation (following Long et al. 2019) and maintained on potato dextrose agar (PDA) and oatmeal agar (OA) media under aseptic conditions, at 25 °C for 1–5 weeks.

### DNA extraction, PCR amplification, and sequencing

No ascospore germination was observed on potato dextrose agar (PDA) or oatmeal agar (OA), therefore, genomic DNA was extracted directly from the perithecial contents using the BIOMIGA Fungus Genomic DNA Extraction Kit following the manufacturer’s protocol. DNA samples were stored at –20 °C. The internal transcribed spacer (ITS) region, the large subunit (LSU) of ribosomal DNA, the β-tubulin (*tub2*), and the second largest subunit of RNA polymerase II (*rpb2*) genes were amplified by polymerase chain reaction (PCR) using primer pairs ITS1/ITS4 (White et al. 1990; Gardes and Bruns 1993), LR0R/LR5 (Vilgalys and Hester 1990), Bt2a/Bt2b (Glass and Donaldson 1995), and rpb2-5f/7cR (Liu et al. 1999), respectively. The PCR reaction mixture (25 μL total volume) contained 9.5 μL of double-distilled water, 12.5 μL of PCR Master Mix, 1 μL of each forward and reverse primer (10 μM), and 1 μL of template DNA. The amplification followed the protocol of Samarakoon et al. (2022). Amplified PCR products were examined through 1.5% agarose gel electrophoresis, stained with GoldenView, and sent to Sangon Co., China, for sequencing.

### Sequence alignment and phylogenetic analyses

All newly generated sequences were deposited in GenBank (Table 1). Preliminary identification was performed by comparing the sequences with those in the GenBank database using the BLASTN algorithm. Phylogenetic analyses were conducted based on a combined dataset of ITS, LSU, *tub2* and *rpb2* sequences. Reference sequences were retrieved from recent publications and from BLASTN results showing high similarity. Sequences were aligned using MAFFT v.7.110 (Katoh et al. 2019) with default settings; respective alignments were adjusted manually using BioEdit v.7.0.5.3 (Hall 1999) where necessary. The ML analysis was conducted using RAxML v.8.2.12, employing the GTRGAMMA substitution model with 1,000 bootstrap replicates (Stamatakis 2014). Bayesian inference phylogenetic analyses were performed using MrBayes v. 3.2.1 (Ronquist et al. 2012, online version). The model of evolution was estimated by MrModeltest 2.2 (Nylander 2004). The Markov chain Monte Carlo (MCMC) sampling in MrBayes v.3.2.2 (Ronquist et al. 2012) was used to determine the posterior probabilities (PP). Six simultaneous Markov chains were run for 1,000,000 generations, and trees were sampled every 1000^th^ generation. The phylogenetic tree was visualized in FIGTREE v.1.4.3 (Rambaut 2018). All analyses were run on the CIPRES Science Gateway v 3.3 web portal (Miller et al. 2010).

**Table 1.** Taxa and corresponding GenBank accession numbers of sequences used in the phylogenetic analysis.

**Species**	**Strain number**	**GenBank accession numbers**				**References**
		**ITS**	**LSU**	***rpb2***	***tub2***	
*Anthostomella leucobasis*	GMB1143^T^	PP153382	NA	PP198092	NA	Li et al. (2024)
*Anthostomella vestita*	GMB1152^T^	PP153388	NA	NA	NA	Li et al. (2024)
*Barrmaelia oxyacanthae*	CBS 142770	MF488988	MF488997	NA	MF489016	Voglmayr et al. (2018)
*Barrmaelia rhamnicola*	CBS 142772^T^	MF488991	MF489000	NA	MF489019	Voglmayr et al. (2018)
*Minuticlypeus biconcavus*	GMB6221^T^	PQ874038	PQ860484	NA	NA	Habib et al. (2025)
*Minuticlypeus biconcavus*	GMB6222	PQ874039	PQ860485	NA	NA	Habib et al. (2025)
*Minuticlypeus discosporus*	HHUF 30673^T^	LC760559	LC760579	NA	NA	Sugita et al. (2024)
*Minuticlypeus discosporus*	HHUF 30672	LC760558	LC760578	LC760598	NA	Sugita et al. (2024)
*Minuticlypeus rhaphidophylli*	GMB1150	PP153386	PQ860487	NA	PP203034	Habib et al. (2025)
*Minuticlypeus xiaoheensis*	GMB4503^T^	PQ066510	PQ066518	NA	PQ083530	Zhou et al. (2024)
*Minuticlypeus yunnanensis*	GMB5631^T^	PQ884705	PQ885417	NA	PQ893602	Liu et al. (2025)
*Minuticlypeus yunnanensis*	GMB5638	PQ884706	PQ885418	NA	NA	Liu et al. (2025)
*Minuticlypeus zunyiensis*	GMB7604^T^	PZ239397	PZ575029	NA	PZ686047	This study
*Minuticlypeus zunyiensis*	GMB7704	PZ239398	PZ575030	NA	PZ686048	This study
*Nigropunctata bambusicola*	MFLU 19-2134^T^	MW240662	MW240592	MW658644	NA	Samarakoon et al. (2022)
*Nigropunctata bambusicola*	MFLU 19-2145^T^	NR175684	NG081505	MW658646	NA	Samarakoon et al. (2022)
*Nigropunctata chinensis*	GMB6223^T^	PQ874034	PQ860480	PQ826932	PQ863998	Habib et al. (2025)
*Nigropunctata chinensis*	GMB6224	PQ874035	PQ860481	PQ826933	PQ863999	Habib et al. (2025)
*Nigropunctata chiangraiensis*	MFLUCC23-0238^T^	OR909712	NA	OR757300	NA	Tun et al. (2024)
*Nigropunctata complanate*	HHUF 30675^T^	NR190923	NG243035	LC760600	NA	Sugita et al. (2024)
*Nigropunctata complanate*	HHUF 30677	LC760563	LC760583	LC760602	NA	Sugita et al. (2024)
*Nigropunctata hydei*	MC22-020^T^	OR507150	OR507163	OR504422	NA	Samarakoon et al. (2023)
*Nigropunctata khalidii*	GMB1156^T^	PP153389	NA	NA	PP209114	Li et al. (2024)
*Pallidoperidium chinense*	GMB6227^T^	PQ874032	PQ860478	NA	PQ863996	Habib et al. (2025)
*Pallidoperidium chinense*	GMB6228	PQ874033	PQ860479	NA	PQ863997	Habib et al. (2025)
*Pallidoperidium exasperatum*	HHUF 30174^T^	NR190924	NG243036	LC760603	NA	Sugita et al. (2024)
*Pallidoperidium exasperatum*	HHUF 30667	LC760567	LC760587	NA	NA	Sugita et al. (2024)
*Pallidoperidium paraexasperatum*	HHUF 30668^T^	NR190925	NG243037	NA	NA	Sugita et al. (2024)
*Pallidoperidium smilacis*	GMB1151	PP153387	PQ860486	NA	PP203035	Habib et al. (2025)
*Pallidoperidium yunnanense*	GMB6414^T^	PX782162	PX782156	PX789095	NA	He et al. (2026)
*Pallidoperidium yunnanense*	GMB6415	PX782161	PX782155	PX789096	NA	He et al. (2026)
*Paravamsapriya nypae*	SNT88	PP592459	PP621087	PP780256	PP816197	Zhang et al. (2024)
*Paravamsapriya nypae*	SNT155	PP592460	PP621088	PP780257	NA	Zhang et al. (2024)
*Paravamsapriya ostiolata*	MFLU 18-0761	MW240624	MW240553	NA	MW775581	Samarakoon et al. (2022)
*Paravamsapriya ostiolata*	MFLU 18-0813	MW240625	MW240554	NA	MW775582	Samarakoon et al. (2022)
*Paravamsapriya yunnanensis*	GMB7613^T^	PZ239399	PZ575031	NA	PZ686049	This study
*Paravamsapriya yunnanensis*	GMB7713	PZ239400	PZ575032	NA	PZ686050	This study
*Pseudoanthostomella pini-nigrae*	MFLUCC 16-0478	KX533453	KX533454	NA	NA	Samarakoon et al. (2022)
*Pseudoanthostomella senecionicola*	MFLUCC 15-0013^T^	MW240674	MW240604	MW658653	NA	Samarakoon et al. (2022)
*Vamsapriya bambusicola*	MFLUCC 11-0477	KM462835	KM462836	KM462834	KM462833	Dai et al. (2014)
*Vamsapriya bambusicola*	MFLUCC 11-0637	KU940159	KU863147	KU940182	NA	Dai et al. (2014)
*Vamsapriya indica*	MFLUCC 12-0544^T^	KM462839	KM462840	KM462841	KM462838	Dai et al. (2014)
*Vamsapriya indica*	MFLUCC 21-0088	MZ613172	MZ613169	OK560921	NA	Sun et al. (2021)
*Vamsapriya kailiensis*	GMB6237	PQ874043	PQ860492	NA	PQ864006	Habib et al. (2025)
*Vamsapriya kailiensis*	GMB6236^T^	PQ874042	PQ860491	NA	PQ864005	Habib et al. (2025)
*Vamsapriya khunkonensis*	MFLUCC 11-0475	KM462830	KM462831	KM462829	KM462828	Dai et al. (2014)
*Vamsapriya mucosa*	MFLU 18-0103^T^	MW240622	MW240551	MW658614	MW775580	Samarakoon et al. (2022)
*Vamsapriya yunnana*	KUMCC 18-0008^T^	MG833874	MG833873	MG833875	NA	Jiang et al. (2022)

Notes: Type specimens are marked with T; “N/A”: indicates no sequence available in GenBank. Newly generated sequences are shaded in blue.

### Registration of novel taxa

Index Fungorum Registration Identifiers were obtained for the new species as mentioned in Index Fungorum (2026).

## Results

### Phylogeny

**Figure 1.**
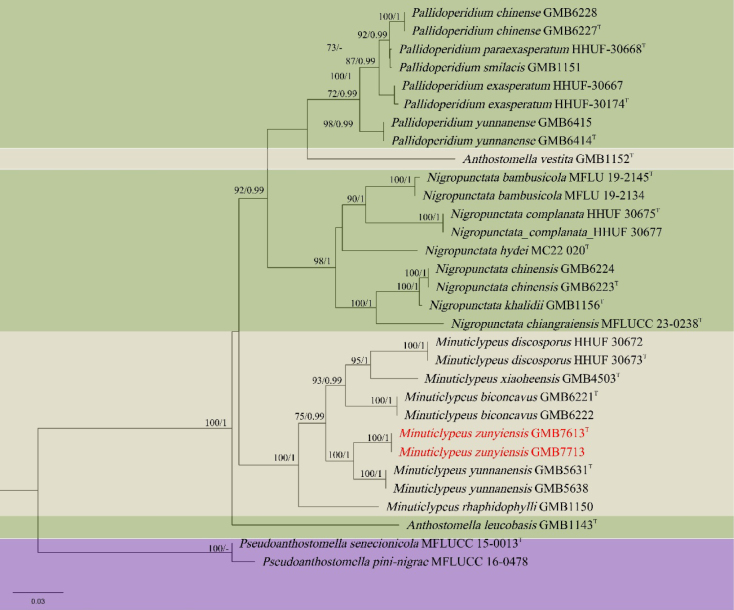
RAxMLtree of *Minuticlypeus* species and related taxa based on combined ITS, LSU, *tub2*, and *rpb2* sequences. Bootstrap support values for maximum likelihood (ML) greater than 70% and Bayesian posterior probabilities (BPP) greater than 0.90 are displayed above the respective branches (ML/BI). The best-scoring RAxML tree showed a final maximum likelihood optimization value of −12274.915712. Estimated base frequencies were as follows: A = 0.247642, C = 0.245222, G = 0.265487, and T = 0.241649. Substitution rates were AC = 1.153534, AG = 3.174185, AT = 1.383242, CG = 0.820682, CT = 6.338643, and GT = 1.000000. The gamma distribution shape parameter alpha was 0.884372, and the tree length was 1.126155. Type materials are marked with “T”, and the strains of newly described species are highlighted in red.

The aligned dataset used for phylogenetic reconstruction of *Minuticlypeus* species (Fig. 1) consisted of 3,049 characters after the exclusion of ambiguously aligned regions and long gaps (ITS 1–485; LSU 486–1586; *tub2* 1587–2052; and *rpb2* 2053–3049). *Barrmaelia oxyacanthae* and *B. rhamnicola* were chosen as outgroup taxa. In the phylogram (Fig. 1), species of *Minuticlypeus* form a well-supported monophyletic clade. The sequences generated in the present study for *Minuticlypeus zunyiensis* formed a well-supported, distinct clade sister to *M. yunnanensis* (100% BS/1.0 PP; Fig. 1).

**Figure 2.**
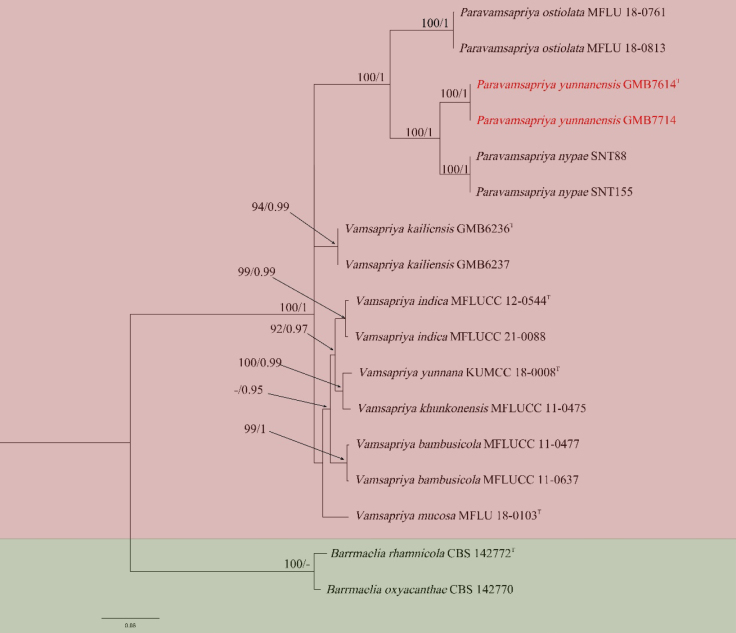
RAxML tree of *Paravamsapriya* species and related taxa based on combined ITS, LSU, *tub2*, and *rpb2* sequences. Bootstrap support values for maximum likelihood (ML) greater than 70% and Bayesian posterior probabilities (BPP) greater than 0.90 are displayed above the respective branches (ML/BI). The best-scoring RAxML tree showed a final maximum likelihood optimization value of −10564.663219. Estimated base frequencies were as follows: A = 0.237599, C = 0.264539, G = 0.267435, and T = 0.230428. Substitution rates were AC = 0.980267, AG = 2.581063, AT = 0.978201, CG = 0.830856, CT = 4.888409, and GT = 1.000000. The gamma distribution shape parameter alpha was 0.485842, and the tree length was 1.577296. Type materials are marked with “T”, and the strains of newly described species are highlighted in red.

The aligned dataset used for phylogenetic reconstruction of the *Paravamsapriya* species (Fig. 2) comprised 2,799 characters after excluding ambiguously aligned regions and long gaps (ITS 1–527; LSU 528–1304; *tub2* 1305–2046; and *rpb2* 2047–2799). *Pseudoanthostomella pini-nigrae* and *P. senecionicola* were chosen as outgroup taxa in Fig. 2. In the phylogram (Fig. 2), species of *Paravamsapriya* form a well-supported distinct clade. The sequences generated in the present study for *Paravamsapriya yunnanensis* formed a well-supported sister clade, with *P. nypae* (100% BS/1.0 PP).

### Taxonomy

#### Minuticlypeus zunyiensis

Taxon classificationFungiXylarialesPallidoperidiaceae

Z.Q. Yao, K. Habib & Q.R. Li sp. nov.

4DC266AE-9A09-5341-9F41-6D6B324CD7E2

Fig. 3

##### Etymology.

The specific epithet ‘*zunyiensis*’ refers to Zunyi City, Guizhou Province, China, where the holotype specimen was collected.

**Figure 3.**
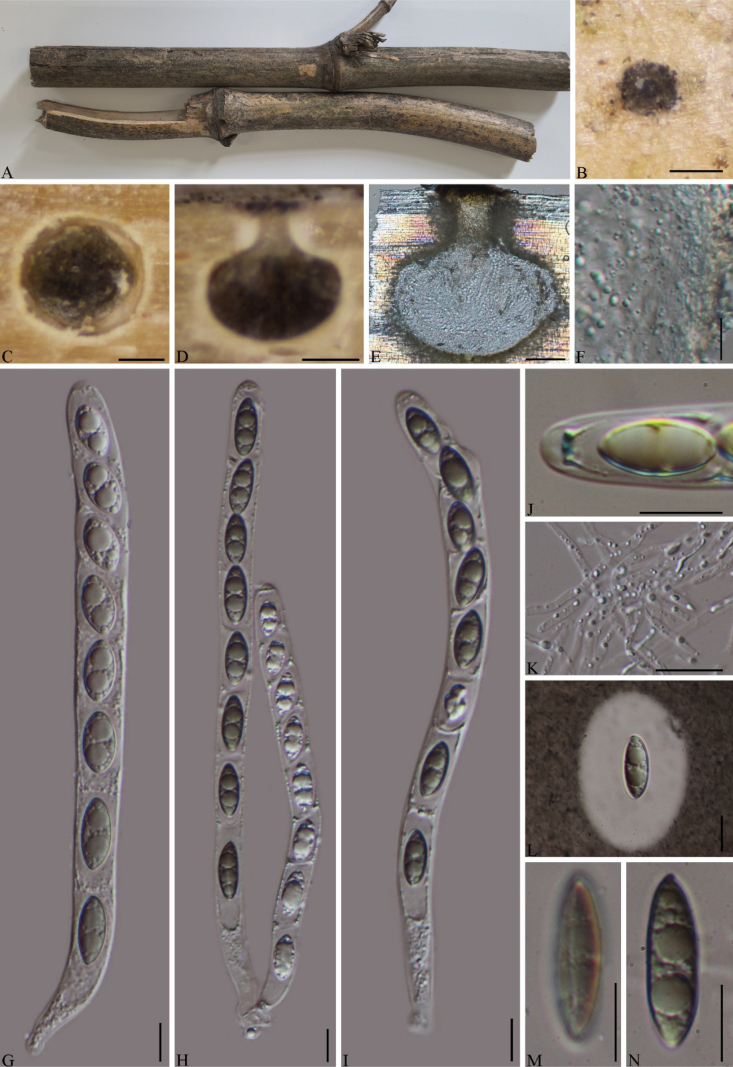
*Minuticlypeus zunyiensis* (GMB7604, holotype). **A**. Bamboo hosts; **B, C**. Ascomata on the surface of host; **D, E**. Longitudinal sections of ascomata; **F**. Peridium; **G–I**. Asci in distilled water; **J**. J+ apical ring; **K**. Paraphyses; **L–N**. Ascospores (**L**. In Indian ink). Scale bars: 0.2 mm (**B–D**); 100 µm (**E**); 10 µm (**F–N**).

##### Holotype.

China • Guizhou Province, Zunyi city, Kuankuoshui National Nature Reserve (28°6'25.65"N, 107°2'20.67"E), altitude 1366 m, on dead culms of unidentified bamboo species, 22 August 2025, Z.Q. Yao, 2025KKS5 (GMB7604, holotype); • ibid. KUN-HKAS 154873, isotype.

##### Description.

Saprobic on dead bamboo culms, forming black, circular spots on the host surface. **Sexual morph: *Ascomata*** immersed, scattered, subglobose, appearing as black, raised dots on the host surface, 354–665 × 407–566 µm (x̄ = 492 × 471.5 µm, n = 15), clypeate, ostiolate. ***Ostiole*** central. ***Peridium*** 7.5–15 µm wide (x̄ = 11.5 µm, n = 15), composed of multiple layers of polygonal, dark brown cells, inner layer hyaline. ***Paraphyses*** 2.3–4.8 µm wide (x̄ = 3.4 µm, n = 15), hyaline, numerous, unbranched, aseptate, containing white cytoplasmic inclusions. ***Asci*** 166.5–208.5 × 7.5–11 µm (x̄ = 184 × 9.5 µm, n = 20), 8-spored, unitunicate, cylindrical, short-pedicellate. Apically 1.3–2.5 × 3.2–4.7 µm (x̄ = 1.9 × 3.9 µm, n = 20) rounded, with a wedge-shaped, J+ (amyloid) apical ring. ***Ascospores*** 16.5–21.5 × 6.5–8.5 µm (x̄ = 18.2 × 7.2 µm, n = 20), uniseriate, fusiform to ellipsoidal, tapering towards both ends with slightly sharp ends, smooth, with a straight to slightly curved, spore-length germ slit, surrounded by a 5–12.6 µm thick mucilaginous sheath. **Anamorph**: Undetermined.

##### Paratype.

China • Guizhou Province, Zunyi city, Kuankuoshui National Nature Reserve (28°6'25.69"N, 107°2'20.75"E), altitude 1388 m, on dead culms of unidentified bamboo species, 22 August 2024, Z.Q. Yao, 2024KKS260 (GMB7704; Paratype).

##### Notes.

In the phylogram (Fig. 1), *Minuticlypeus zunyiensis* forms a well-supported clade (BS = 100; PP = 1.00) in a sister relationship with *M. yunnanensis*. Both species share key morphological features, including an amyloid, discoid apical apparatus, as well as similarly sized asci and ascospores. However, they can be readily distinguished by differences in ascospore shape and in the size of asci and ascomata. *Minuticlypeus zunyiensis* possesses fusiform to ellipsoidal ascospores that taper toward both ends, with slightly pointed tips and a distinct germ slit. In contrast, *M. yunnanensis* has ellipsoid to rugby-shaped ascospores with more rounded ends and lacks a germ slit. Additionally, *M. zunyiensis* has larger ascomata (354–665 × 407–566 µm) compared to *M. yunnanensis* (301–342.4 × 351.6–489.9 µm) (Li et al. 2024).

Compared with other species in the genus, *M. zunyiensis* is also clearly distinguishable (Table 2). *Minuticlypeus discosporus* differs by having smaller ascomata (290–410 × 310–430 µm), much smaller asci (105–130 µm vs. 166.5–208.5 µm), and ellipsoid to oblong ascospores with rounded ends rather than tapering ends (Sugita et al. 2024). *Minuticlypeus rhaphidophylli* also has smaller ascomata (304–336 × 155–183 µm) and shorter asci (133–163 µm) (Li et al. 2024). *Minuticlypeus xiaoheensis* is characterized by smaller ascomata (320–380 × 340–400 µm), shorter asci (85.5–140 µm), and ellipsoid to broadly ellipsoid ascospores with rounded ends (Zhou et al. 2024). *Minuticlypeus biconcavus* differs in having smaller ascomata (300–450 µm wide, 250–350 µm high), shorter asci with an edge-shaped apical apparatus that becomes thinner in the center, forming a biconcave shape, and ascospores lacking a germ slit (Habib et al. 2025).

**Table 2.** Comparison of the main morphological characteristics of the species of *Minuticlypeus*.

**Species**	**Ascomata (µm)**	**Asci (µm)**	**Ascospores (µm)**	**Mucilaginous sheath (µm)**	**Germ Slit**	**Reference**
*M. biconcavus*	300–450 × 250–350	140–175 × 15–25	12–20 × 7–11.5	4–6.5	lack	Habib et al. (2025)
*M. discosporus*	290–410 × 310–430	105–130 × 12.5–16.5	15–20.5 × 7.5–10.5	lack	present	Sugita et al. (2024)
*M. rhaphidophylli*	304–336 × 155–183	133.5–163 × 10.6–16.5	17.8–22.8 × 7.1–8.5	7.2–9.4	present	Habib et al. (2025)
*M. xiaoheensis*	320–380 × 340–400	85.5–140 × 11–18.5	11–21 × 6.5–10.5	5–8	present	Zhou et al. (2024)
*M. yunnanensis*	301–342 × 351–489	149.9–202.4 × 10.7–13.3	19.3–22.4 × 7.8–11.3	4.2–6	lack	Liu et al. (2025)
*M. zunyiensis*	354–665 × 407–566	166.5–208.5 × 7.5–11	16.5–21.5 × 6.5–8.5	5–12.6	present	This study

On the basis of these clear morphological distinctions from all known species of the genus together with its distinct phylogenetic placement, *Minuticlypeus zunyiensis* is herein introduced as a new species.

#### Paravamsapriya yunnanensis

Taxon classificationFungiXylarialesVamsapriyaceae

Z.Q. Yao, K. Habib & Q.R. Li sp. nov.

1427FE67-4BCC-5239-B7F5-80DA5948D754

Fig. 4

##### Etymology.

The specific epithet ‘yunnanensis’ refers to the Yunnan Province, China, where the holotype was collected.

**Figure 4.**
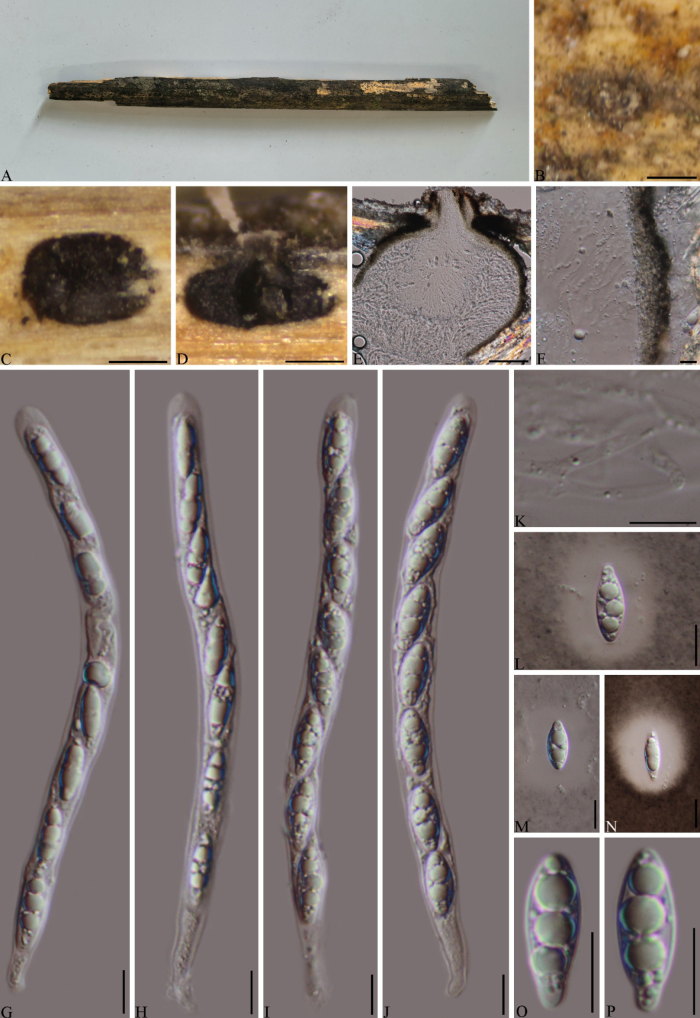
*Paravamsapriya yunnanensis* (GMB7613, holotype). **A**. A bamboo host; **B, C**. Ascomata on the surface of host; **D, E**. Longitudinal sections of ascomata; **F**. Peridium; **G–J**. Asci in distilled water; **K**. Paraphyses; **L–P**. Ascospores (**L–N**. In Indian ink). Scale bars: 0.2 mm (**B–D**); 100 µm (**E**); 10 µm (**F–P**).

##### Holotype.

China • Yunnan Province, Xishuangbanna city, Xishuangbanna National Nature Reserve (24°2'24.61"N, 100°48'38.30"E), altitude 1190 m, on dead culms of unidentified bamboo species 22 September 2025, Z.Q. Yao, 2025XSBN17 (GMB7613, holotype); • ibid. KUN-HKAS 154874, isotype.

##### Description.

Saprobic on dead bamboo culms, forming black, circular spots on the host surface. **Sexual morph: *Ascomata*** 536–622 × 386–438 µm (x̄ = 594 × 409 µm, n = 15), immersed, solitary to aggregated, subglobose, appearing as black dots on the host surface with a central ostiole, subglobose, clypeate, ostiolate. ***Ostiole*** central. ***Peridium*** 11–22.2 µm wide, composed of multiple layers, outer layer comprising yellowish brown, thick-walled, compact cells of ***textura angularis***, inner layer composed of hyaline, thin-walled cells of ***textura angularis***. ***Paraphyses*** 1.7–4.8 µm wide, filiform, aseptate, tapering toward the apex, guttulate, embedded in a gelatinous matrix. ***Asci*** 145–183 × 9–14 µm (x̄ = 162 × 11.5 µm, n = 20), 8-spored, unitunicate, cylindrical, short-pedicellate, apically rounded, with an inconspicuous, J- (non-amyloid), disc-shaped, subapical ring. ***Ascospores*** 17–21.5 × 5.7–7.8 µm (x̄ = 19 × 7 µm, n = 20), uniseriate, ellipsoidal to fusiform, aseptate, hyaline, with a smaller, nodulose basal cell about 1/6 the size of the larger apical cell, surrounded by a 10–31 µm thick mucilaginous sheath, smooth- walled, finely guttulate. **Anamorph**: Undetermined.

##### Paratype.

China • Yunnan Province, Xishuangbanna city, Xishuangbanna National Nature Reserve (24°2'24.63"N, 100°48'38.38"E), altitude 1220 m, on dead culms of unidentified bamboo species, 22 September 2025, Z.Q. Yao, 2024XSBN190 (GMB7713; Paratype).

##### Notes.

In the phylogram (Fig. 2), *Paravamsapriya yunnanensis* forms a well-supported, distinct clade that is sister to *P. nypae* (BS = 100; PP = 1.00). Morphologically, the two species are similar in ascospore and asci characteristics, however, they differ in habitat, ascomatal and ascus dimensions. *Paravamsapriya yunnanensis* was collected on dead bamboo culms (Poaceae), whereas *P. nypae* occurs on palm rachides of *Nypa fruticans*. In addition, *P. yunnanensis* has larger ascomata (536–622 × 386–438 µm) and longer asci (145–183 µm) than *P. nypae*, which has ascomata 200–360 μm diam., and asci 90–145 µm long (Zhang et al. 2024). When compared with the type species of the genus, *P. ostiolata*, the new species is clearly distinguishable by its smaller ascospores (17–21.5 × 5.7–7.8 µm) surrounded by a relatively thick mucilaginous sheath (10–31 µm). In contrast, *P. ostiolata* possesses larger ascospores (23–28 × 7.5–10 µm) with prominent thin mucilaginous caps at both ends, which are absent in *P. yunnanensis* (Samarakoon et al. 2022).

Based on these clear morphological distinctions within the genus, together with its distinct phylogenetic placement, *P. yunnanensis* is herein introduced as a new species.

### Key to species of *Paravamsapriya*

**Table:** 

1	Ascospores 23–28 × 7.5–10 μm, with mucilaginous cap at both ends	***P. ostiolata***
–	Ascospores < 23 µm, lack a mucilaginous cap at both ends	**2**
2	On palm rachides of *Nypa fruticans*, asci 90–145 µm in length	***P. nypae***
–	On bamboo culms, asci 145–183 µm in length	***P. yunnanensis***

## Discussion

In this study, two new species, *Minuticlypeus zunyiensis* and *Paravamsapriya yunnanensis*, are described from bamboo substrates in China. Both genera show a strong ecological preference for monocotyledonous hosts, particularly bamboo. To date, all known species of *Minuticlypeus* have been reported from bamboo, while within *Paravamsapriya*, two species (including the newly described *P. yunnanensis* and *P. ostiolata*) occur on bamboo and one (*P. nypae*) is known from palm rachides (Samarakoon et al. 2022; Li et al. 2024; Sugita et al. 2024; Zhang et al. 2024; Habib et al. 2025). The report of two additional bamboo-associated species in this study indicates that these genera are ecologically adapted to monocot hosts, particularly Poaceae and Arecaceae, and emphasizes the importance of exploring understudied bamboo microhabitats as reservoirs of fungal diversity.

Phylogenetically, species of both genera form well-supported monophyletic clades, confirming their taxonomic placement and evolutionary distinctiveness. However, the phylogenetic relationships within these clades do not always correspond closely with morphological similarities. For example, *M. yunnanensis* and *M. biconcavus* both lack a germ slit in their ascospores, yet they are not closely related in the phylogenetic analyses. Similarly, *M. zunyiensis* and *M. rhaphidophylli* share similar ascospore morphology, characterized by fusiform to ellipsoidal shapes tapering toward both ends with slightly pointed tips, but are phylogenetically not closely related. *M. biconcavus* is particularly distinctive in the genus due to its unique wedge-shaped apical apparatus that becomes thinner in the center, forming a characteristic biconcave structure. This morphological–phylogenetic incongruence may reflect limited taxon sampling, convergent evolution of ascospore traits under similar ecological pressures, or retention of ancestral character states that obscure true evolutionary relationships (Habib et al. 2025; Liu et al. 2025).

In *Paravamsapriya*, the new species *P. yunnanensis* and its sister taxon *P. nypae* are both morphologically and phylogenetically well separated from the type species *P. ostiolata*. While *P. ostiolata* possesses ascospores with pointed ends and thin, slightly curved mucilaginous caps at both poles, the two sister species have ascospores with broader mucilaginous sheaths and more rounded ends, clearly distinguishing them from the type species (Samarakoon et al. 2022; Zhang et al. 2024).

The clear morphological distinctions from all known congeners, together with their well-supported and distinct phylogenetic placements, unambiguously justify the introduction of *Minuticlypeus zunyiensis* and *Paravamsapriya yunnanensis* as two novel species. These findings further broaden current knowledge of species diversity, substrate specificity, and morphological evolution within bamboo-associated fungi, particularly among Xylariales lineages. Moreover, they highlight the ecological and evolutionary significance of bamboo as an important reservoir for fungal diversification.

## Supplementary Material

XML Treatment for Minuticlypeus zunyiensis

XML Treatment for Paravamsapriya yunnanensis

## Additional information

### Artificial Intelligence (AI) use

The authors accept full responsibility for the content of the manuscript, including the disclosure of any use of AI.

No AI tools were used in the preparation of this manuscript.

### Author ORCIDs

Z.-Q. Yao https://orcid.org/0009-0001-9434-1951

L.-L. Liu https://orcid.org/0009-0003-8481-6137

W.-H. Li https://orcid.org/0009-0004-8845-0969

Q. Yang https://orcid.org/0009-0006-2794-5583

N.N. Wijayawardene https://orcid.org/0000-0003-0522-5498

A.M. Elgorban https://orcid.org/0000-0003-3664-7853

F.Z. Filimban https://orcid.org/0000-0001-7527-1313

K. Habib https://orcid.org/0000-0003-2572-0306

Q.-R. Li https://orcid.org/0000-0001-8735-2890

### Data availability

All of the data that support the findings of this study are available in the main text.
